# β-Adrenergic Receptor Stimulation and Alternans in the Border Zone of a Healed Infarct: An *ex vivo* Study and Computational Investigation of Arrhythmogenesis

**DOI:** 10.3389/fphys.2019.00350

**Published:** 2019-03-29

**Authors:** Jakub Tomek, Guoliang Hao, Markéta Tomková, Andrew Lewis, Carolyn Carr, David J. Paterson, Blanca Rodriguez, Gil Bub, Neil Herring

**Affiliations:** ^1^Department of Computer Science, British Heart Foundation Centre of Research Excellence, University of Oxford, Oxford, United Kingdom; ^2^Department of Physiology, Anatomy and Genetics, British Heart Foundation Centre of Research Excellence, University of Oxford, Oxford, United Kingdom; ^3^Nuffield Department of Medicine, University of Oxford, Oxford, United Kingdom; ^4^Department of Physiology, McGill University, Montreal, QC, Canada

**Keywords:** myocardial infarction, β-adrenergic receptor, sympathetic nervous system, alternans, arrhythmias

## Abstract

**Background:** Following myocardial infarction (MI), the myocardium is prone to calcium-driven alternans, which typically precedes ventricular tachycardia and fibrillation. MI is also associated with remodeling of the sympathetic innervation in the infarct border zone, although how this influences arrhythmogenesis is controversial. We hypothesize that the border zone is most vulnerable to alternans, that β-adrenergic receptor stimulation can suppresses this, and investigate the consequences in terms of arrhythmogenic mechanisms.

**Methods and Results:** Anterior MI was induced in Sprague-Dawley rats (*n* = 8) and allowed to heal over 2 months. This resulted in scar formation, significant (*p* < 0.05) dilation of the left ventricle, and reduction in ejection fraction compared to sham operated rats (*n* = 4) on 7 T cardiac magnetic resonance imaging. Dual voltage/calcium optical mapping of post-MI Langendorff perfused hearts (using RH-237 and Rhod2) demonstrated that the border zone was significantly more prone to alternans than the surrounding myocardium at longer cycle lengths, predisposing to spatially heterogeneous alternans. β-Adrenergic receptor stimulation with norepinephrine (1 μmol/L) attenuated alternans by 60 [52–65]% [interquartile range] and this was reversed with metoprolol (10 μmol/L, *p* = 0.008). These results could be reproduced by computer modeling of the border zone based on our knowledge of β-adrenergic receptor signaling pathways and their influence on intracellular calcium handling and ion channels. Simulations also demonstrated that β-adrenergic receptor stimulation in this specific region reduced the formation of conduction block and the probability of premature ventricular activation propagation.

**Conclusion:** While high levels of overall cardiac sympathetic drive are a negative prognostic indicator of mortality following MI and during heart failure, β-adrenergic receptor stimulation in the infarct border zone reduced spatially heterogeneous alternans, and prevented conduction block and propagation of extrasystoles. This may help explain recent clinical imaging studies using meta-iodobenzylguanidine (MIBG) and 11C-meta-hydroxyephedrine positron emission tomography (PET) which demonstrate that border zone denervation is strongly associated with a high risk of future arrhythmia.

## Introduction

Sudden cardiac death (SCD) due to ventricular arrhythmias is one of the leading causes of mortality worldwide, and patients with a history of myocardial infarction (MI) are at particularly high risk (Soo et al., 2001). Infarcted hearts undergo considerable remodeling, providing a substrate for arrhythmia via multiple mechanisms. Firstly, the structure of the scar is heterogeneous with thin, slow-conducting fibers of myocytes able to promote re-entry (Richardson et al., 2015). This is exacerbated by changes in the expression of membrane ion channels and under-expression and reorganization of gap junctions (Luke and Saffitz, 1991) in the infarct border zone also slowing conduction (Tse, 2016). Secondly, remodeling of ion channels, transporters and calcium-calmodulin dependent protein kinase II (CaMKII) signaling influences calcium handling and can predispose the myocardium to enhanced automaticity, thereby providing an arrhythmic trigger (Bogun et al., 2008). Thirdly, the sympathetic innervation of the infarct border zone also undergoes significant remodeling (Li and Li, 2015; Habecker et al., 2016), with infarct denervation occurring post-MI, followed by subsequent reinnervation (Cao et al., 2000a,b). This may be accompanied by β-adrenergic receptor super-sensitivity (Gardner et al., 2015), or downregulation (Karliner et al., 1986).

While high levels of overall sympathetic drive are clearly a negative prognostic indicator of mortality following MI and during heart failure (Ardell et al., 2016; Shivkumar et al., 2016), the exact contribution of localized sympathetic reinnervation and β-adrenergic receptor stimulation in the infarct border zone is controversial. Animal research suggests that reinnervation of the border zone may be pro-arrhythmic (Cao et al., 2000a; Shen and Zipes, 2014). However, multiple recent clinical imaging studies using the norepinephrine analog meta-iodobenzylguanidine (ADMIRE-HF, Jacobson et al., 2010) and 11C-meta-hydroxyephedrine (PARAPET, Fallavollita et al., 2014) positron emission tomography (PET), report sympathetic denervation following MI as a strong predictor of ventricular arrhythmias and SCD. This is a striking result although highly surprising and may appear mechanistically counter-intuitive.

Episodes of fibrillation are also observed to be consistently preceded by ‘alternans’ formation, the beat-by-beat alternation of long and short repolarization durations (Wilson et al., 2009). Post-MI patients are more vulnerable to repolarization alternans compared to non-infarcted patients (Koller et al., 2005), and alternans is an independent predictor of SCD after MI (Yu et al., 2012). Importantly, β-adrenergic receptor activation is known to modulate cellular calcium handling and may attenuate the generation of alternans in a ventricular cell model (Tomek et al., 2017). Experimental data suggests that while β-adrenergic stimulation attenuates alternans in normal myocardium (Wang et al., 2014), it may exacerbate alternans during acute ischaemia (Nearing et al., 1991). How β-adrenergic stimulation in the border zone of a healed MI influences arrhythmia generation is poorly understood. We therefore tested the hypothesis that β-adrenergic receptor stimulation can attenuate border zone alternans in hearts with a healed MI. We complement experimental studies with computer simulations to reproduce our findings and determine how β-adrenergic receptor stimulation in the border zone region influences arrhythmogenic mechanisms. The combination of experiments and computer simulations overcome experimental limitations and aims to help our understanding of the consequences of specific border zone denervation.

## Materials and Methods

This study was carried out in accordance with the principles of the Basel Declaration, the UK Home Office Guidance on the operation of the Animals (Scientific Procedures) Act 1986 and to institutional guidelines. The protocol was approved by the University of Oxford Animal Care and Ethical Review Committee (PPL 30/3322).

### Myocardial Infarction

Anesthesia was induced in male Sprague-Dawley rats (*n* = 8) fed *ad libitum* weighing 350–500 g using 4% isoflurane in medical oxygen and maintained at 2% isoflurane. The trachea was intubated to ventilate the lungs and a left thoracotomy performed. The heart was exteriorized and a 9 mm aluminum cryoprobe cooled to the temperature of liquid nitrogen applied to the antero-apical myocardium for 15 s. The chest was closed in layers and analgesia provided with meloxicam and buprenorphine. The method of cryoinfarction was adapted from Ciulla et al. (2004) and we have previously compared hearts with damage induced by cryoinjury with those from ischaemia/reperfusion and found a similar pattern of fibrosis and macrophage infiltration, with a more reproducible infarct size (Lewis et al., 2018). The cryoinfarction was followed by a 6- to 8-week healing period, after which magnetic resonance imaging (MRI) was performed. In sham-operated (*n* = 4) animals, no infarction was performed; however, thoracotomy and cardiac exteriorization were performed as in the infarcted animals.

### Magnetic Resonance Imaging

Cardiac structure and function were assessed *in vivo* using cine MRI on a 7 T scanner (Varian Medical Systems, Yarnton, United Kingdom) using a quadrature high pass birdcage volume transmit RF coil with a 72 mm internal diameter (RAPID Biomedical GmbH, Germany) and a phase array 4-channel RF surface receive coil (RAPID Biomedical GmbH, Germany). Anesthesia was induced at 2.5–3% isoflurane in oxygen and nitrous oxide (4:1, total of 2 L/min) and maintained at 2% isoflurane. Animals were placed in a home-built animal handling system. Body temperature was maintained using air heating, and a two-lead electrocardiogram (ECG) for cardiac gating was obtained using leads placed subcutaneously into the upper forelimbs. A series of sagittal and axial scout ECG-gated fast low-angle shot (FLASH) cardiac-localized images were acquired and used to define the axes of the heart following global shimming. Between 10 and 11 contiguous cine-MR images (28–35 frames per cardiac cycle) were acquired of the heart in the short-axis orientation covering the entire heart. Imaging parameters were as given; field of view (FOV): 51.2 × 51.2 mm, matrix size: 192 × 192, slice thickness: 1.6 mm, TE/TR: 1.43/4.6 ms, Gaussian RF excitation pulse: ∼25 degrees and 4 averages. Heart rate remained stable throughout the procedure. End-diastolic (ED) and end-systolic (ES) frames were selected as those with the largest and smallest cavity volumes, respectively, and the maximum dimension was recorded as end systolic and diastolic lumen, respectively. Epicardial and endocardial borders were outlined using the freehand drawing function of ImageJ (National Institutes of Health, United States). Measurements from all slices were summed to calculate ED volume (EDV), ES volume (ESV), stroke volume (SV = EDV - ESV), ejection fraction (EF = SV/EDV). LV mass was calculated as myocardial area × slice thickness × myocardial specific gravity (1.05). Average mass was then computed as the average of LV mass at systole and at diastole. In each MRI slice, the length of akinetic epicardium and the total length of epicardial surface in a slice were recorded as akin_i_ and total_i_ for the i-th slice. Then, relative infarct size was defined as $\sum_{i}\frac{akin_{i}}{total_{i}}$.

Subsequently, rats were euthanized by overdose by pentobarbital (0.3 ml, 100 g) under deep anesthesia (3% isoflurane and 97% oxygen). The hearts were excised and perfused using the Langendorff scheme as described previously (Kalla et al., 2016), and dual voltage and calcium mapping was performed as described below.

### Langendorff Heart and Imaging

**FIGURE 1:**
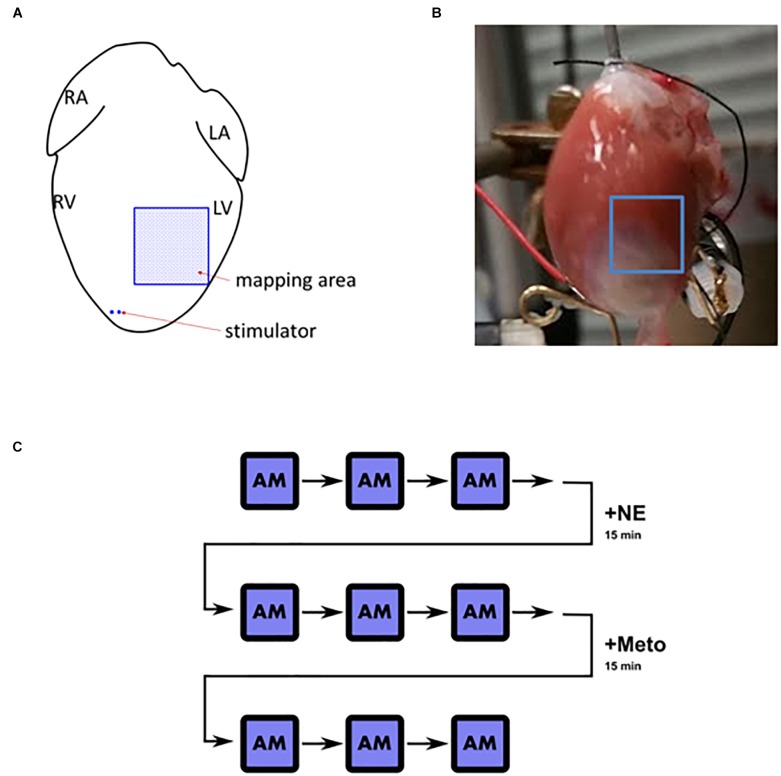
Langendorff heart overview. **(A)** Schematic depiction of the positioning of stimulator and the mapped area (L/R, left/right; A/V, atrium/ventricle). **(B)** An example of the mapped field of view in an infarcted heart. **(C)** A schematic illustration of the pacing and stimulation protocol. AM, alternans mapping; +NE, norepinephrine (1 μmol/L) for 15 min; +Meto, metoprolol (10 μmol/L) for 15 min. After each AM instance, the heart was not stimulated for 2–3 min for the heart rate to stabilize before the following measurement took place.

The hearts were perfused with oxygenated Tyrode solution (flow rate 10 ml/min; NaCl 120.3 mM, KCl 4 mM, MgSO_4_^∗^7H_2_0 1.3 mM, NaH_2_0P_4_ 1.2 mM, CaCl 1.2 mM, NaHCO_3_ 25.2 mM, glucose 11 mM). Blebbistatin (10 μM) was used to stabilize the heart and prevent motion artifacts during imaging and pluronic (100 μl per 200 ml of Tyrode solution) was used to aid dye loading. After the heart stopped moving, a thin plastic tube was inserted to the left ventricle via the left atrial appendage and mitral valve to prevent perfusate pressure build-up. Rhod-2 was used for calcium mapping and RH-237 for voltage mapping (injected through a gel membrane into the plastic tube above the perfusion cannula; 100 μl over 2 min and 20 μl over 1 min, respectively). The imaging system is based on the design by Choi and Salama (2000), but here we use two Photometrics Evolve 128 cameras attached to a dual emission image splitter (Cairn Research TwinCam). Light is collected using a 50 mm f0.95 lens (Navitar), and is directed to the cameras after passing through a dichroic mirror with a cut-off of 630 nm. Fluorescence light below 630 nm passes through a 585 ± 20 nm interference filter and the fluorescence light above 630 nm was passed through a 715 nm high pass filter before being imaged by the cameras. Two green LEDs (530 nm Cairn Research OptoLED) were used to illuminate the heart after being passed through bandpass filters (530 ± 20 nm) to minimize stray excitation light reaching the sensors. Custom written software was used to capture images from both cameras and control the stimulator (Model DS2A, Digitimer Ltd.). The software interfaced with a micro-controller (Arduino Uno) to synchronize excitation lights, both cameras and the stimulator to minimize phototoxicity (excitation lights were switched on for 1 s at the end of a stimulation protocol to capture the results from S1 and S2 stimuli). After hanging the hearts in the way that the FOV contained both the infarct border zone and the adjacent non-infarcted myocardium (Figure 1), a forceps tip easily visible on the microscope was positioned in front of the heart close to its surface and moved within the FOV, observing the myocardium under the forceps, making sure that the FOV indeed contains both areas of tissue. A second validation of the FOV positioning was differential loading of dyes. The positioning was done in a way to exclude the infarct itself (based on the voltage and calcium signals, which were weak in the scar). The position of the non-infarcted-border zone boundary was recorded for future image analysis, when determining which pixels belong to which zone. After dye loading, the Langendorff apparatus was set to recycle perfused Tyrode solution.

The calcium and voltage imaging were performed using an “alternans mapping” protocol. In a single episode of alternans mapping, the heart was consecutively paced at basic cycle lengths (bcl) of 140, 130, 120, 110, 100, 95, 90, 85, 80 ms, pacing for 10 s at each bcl, recording the last second of the condition. Raw spatial resolution was 32-by-32 pixels with total FOV wide 7-by-7 mm and with temporal resolution of 1 KHz. As illustrated in Figure 1C, three episodes of alternans mapping were recorded in control conditions. Subsequently, norepinephrine was added to the Tyrode solution (1 μmol/L, recycled to maintain the concentration), perfused for 15 min, and alternans mapping was repeated in the same heart three times again. Afterward, metoprolol (10 μmol/L, recycled) was added to the Tyrode solution and perfused for 15 min and the same measurements were taken as in previous conditions. We used calcium transient duration shortening to confirm β-adrenergic receptor stimulation by norepinephrine.

### Image Processing Methods

Details of image processing methods are given in the Supplementary Methods. In alternans mapping, alternans magnitude of each pixel was determined separately, ultimately providing spatial information on alternans pattern. To estimate calcium alternans magnitude of a single pixel, *A* was defined as average calcium transient amplitude of even action potentials and *B* as average calcium transient amplitude of odd action potentials. Then, alternans magnitude of the given pixel is $\frac{\left|A-B\right|}{A+B}$, which provides information on alternans, without dependence on absolute fluorescence. Action potential duration (APD) alternans is defined similarly, with *A* and *B* defined based on APDs. In the imaging protocol, three repeats of alternans mapping were repeated for each condition. When measuring total alternans for a given recording, condition, and base cycle length, three alternans scores were obtained separately and then averaged to produce the final number. Calcium transient duration estimation for a recording, condition, and base cycle length were treated similarly.

### Statistics

Data are presented as median [interquartile range], given that most data reported were not normally distributed (assessed using Shapiro–Wilk test). Non-parametric data from two independent groups were compared using the Mann–Whitney *U* test and paired data using the Wilcoxon signed rank test. Correlation between two variables was assessed using the regression slope test and expressed as *R*^2^ value. All significance tests are two-tailed and significance accepted at *p* < 0.05.

### Computational Modeling Methods

In order to investigate the implications of alternans in infarct border zone, we used the canine ventricular myocyte model (Tomek et al., 2017). Details of the model, and information on the simulation protocols used are given in the Supplementary Methods, including a diagram of the model structure (Supplementary Figure S1). The model allows the choice of cellular phenotypes: infarct border zone myocyte (BZ) or a non-infarcted zone myocyte (NZ), as well as β-adrenergic receptor stimulation.

## Results

### Development of Healed Myocardial Infarction

**FIGURE 2:**
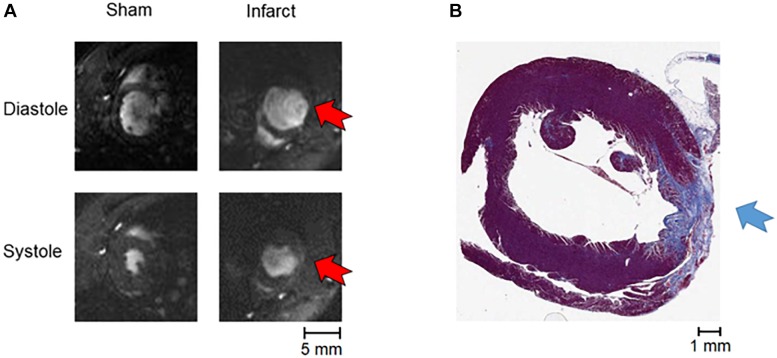
MRI and staining of infarction. **(A)** An example of MRI data (systole and diastole in a sham and an infarcted heart, with red arrows indicating the position of the akinetic infarct). **(B)** An example of Masson’s trichrome staining of apical tissue section showing fibrotic scar formation (blue arrow).

Examples of MRI imaging and Masson’s trichrome staining showing the fibrotic scarring associated with MI are shown in Figure 2. The infarcted hearts manifested significant systolic dysfunction as seen by reduced left ventricular ejection fraction (LVEF) and dilation in the end systolic lumen (Table 1). Given the late stage of infarct healing (2 months after infarction), the developed scars were clearly visible when the hearts were excised, facilitating subsequent detection of the border zone when selecting the region of interest to be imaged; a sample photograph of an infarct is given in Figure 1B.

**Table 1:** Baseline cardiac properties in infarcted and sham hearts as determined by cardiac MRI.

Feature	Infarcted (*n* = 8)	Sham (*n* = 4)	*p*-value
Relative infarct size (%)	6.5 (3.5–9.5)	0 (0–0)	0.004
LVEF (%)	61 (57.5–66)	72 (68.5–77)	0.012
Average mass (mg)	993.5 (959–1047)	853.5 (805.5–922.5)	0.049
End systolic lumen (μl)	266 (216.5–316.5)	153 (140.5–168.25)	0.004
End diastolic lumen (μl)	668.5 (589.5–828)	584 (493.5–672)	0.283
Body weight (g)	493.5 (487.5–513)	492 (455–524)	0.897


*Shown is the median (interquartile range), with the statistical difference between groups assessed using Wilcoxon rank-sum test. LVEF, left ventricular ejection fraction.*

### Rapid Pacing Induces Calcium Alternans

**FIGURE 3:**
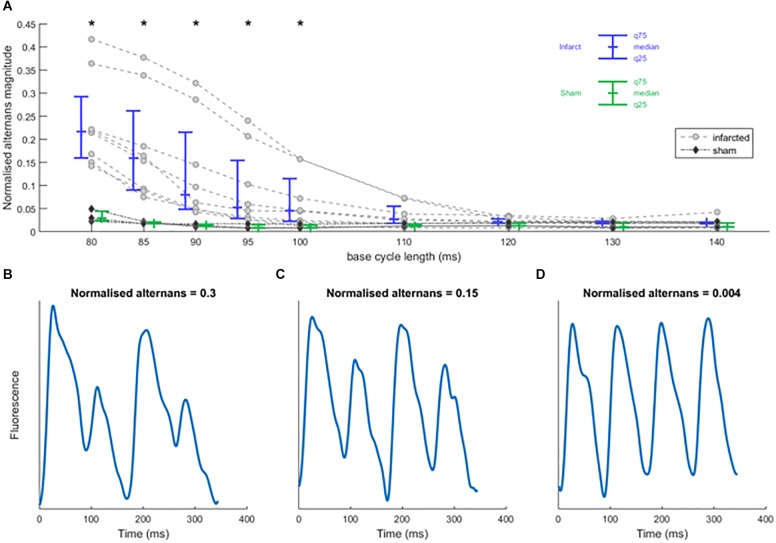
Rapid pacing induces calcium alternans in sham and infarcted hearts. **(A)** Normalized calcium alternans magnitude of recordings in control condition against base cycle length for infarcted (*n* = 8) and sham hearts (*n* = 3). Asterisks represent statistical significance of the difference between sham and infarcted hearts at the level of 0.05 (^∗^); assessed using Mann–Whitney *U* test. **(B–D)** Sample smoothed calcium traces illustrating the values of normalized alternans magnitude.

**FIGURE 4:**
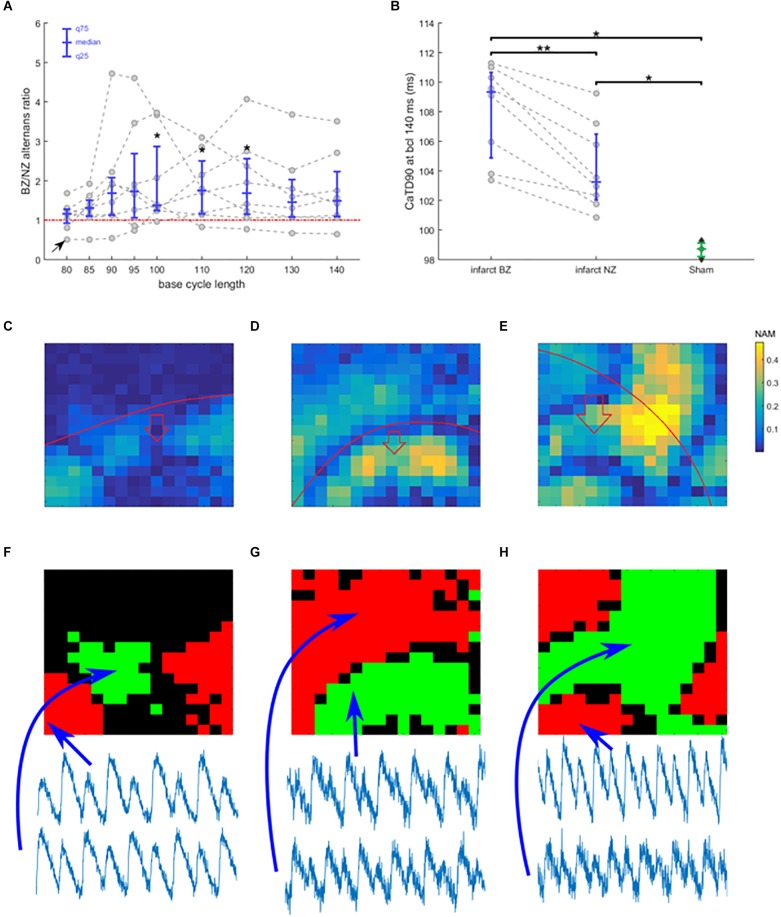
Border zone vulnerability to alternans. **(A)** The ratio of alternans magnitude in border zone and non-infarcted zone of each recording, plotted as gray points against base cycle length (bcl). Data from a single heart are connected with a dashed line. In blue is given the median and 25 and 75 quantiles. Points above the red line manifest more alternans in border zone than in non-infarcted zone. Asterisks represent statistical significance of alternans in non-infarcted zone versus border zone within each heart at the level of 0.05 (^∗^); assessed using the Wilcoxon signed rank test. The black arrow in bottom left corner indicates a special trace discussed further in the text. **(B)** Duration of calcium transient at 90% calcium recovery at bcl 140 in infarcted hearts (NZ, non-infarcted zone; BZ, border zone) and in sham hearts; dashed lines connect data from the same heart. Asterisks represent statistical significance of difference between sham and infarcted hearts at the level of 0.01 (^∗∗^) or 0.05 (^∗^); the Wilcoxon signed rank test was used for comparing BZ and NZ, given the paired nature of data, while for the other two comparisons, Mann–Whitney *U* test was used. **(C)** An example of spatially heterogeneous alternans restricted to BZ. The color in each pixel denotes normalized alternans magnitude (NAM). **(D)** An example of nodal line passing along the edge of BZ. **(E)** An example of nodal line pattern with no obvious relationship to BZ edge. In **(C–E)**, the red line marks the edge of the BZ, with the red arrow showing which side of the red line contains BZ. Subfigures **(C–E)** come from different hearts. **(F–H)** Maps of alternans phase in **(C–E)** (green and red zones represent zones of alternans oscillating in an opposite phase; black zones manifest no alternans). Below the phase maps are shown temporally aligned traces from different zones, verifying the discordance.

In all hearts, increasing the pacing frequency resulted in an increased occurrence of calcium alternans (Figure 3). Infarcted hearts generally manifested visible alternans at bcl up to 100 ms, significantly more so than sham hearts (*p* < 0.05; Wilcoxon signed rank test), which manifested visible alternans only at the shortest bcl of 80 ms. In all infarcted hearts, discordant alternans was observed: a pattern of alternans where two or more zones alternating in the opposite phase are present, separated by lines of no alternans termed “nodal lines” (see example in Figure 4D,E). Discordant alternans is known to be pro-arrhythmic via formation of large repolarization gradients between zones alternating in the opposite phase (Garfinkel, 2007).

As shown in Supplementary Figure S2, we observed a strong correlation between calcium and APD alternans, but as calcium alternans drives APD alternans and could be more accurately measured, further analysis is based on calcium transient alternans.

### The Infarct Border Zone Is More Prone to Alternans Than Non-infarcted Myocardium

The infarct border zone was more prone to alternans, as demonstrated by the ratio of alternans magnitude in border zone versus non-infarcted zone (Figure 4A, example in Figure 3C), with the difference statistically significant for bcls of 100–120 (*p* < 0.05; Wilcoxon signed rank test) and near-significant at 95, 130, and 140 ms (*p* = 0.078, 0.055, 0.078, respectively; Wilcoxon signed rank test). Medium pacing intervals (bcl 100–120 ms) led to the largest increase of alternans in border zone compared to non-infarcted zone; these bcls correspond to the heart rates seen during exercise in rats (Corre et al., 1976). During slower pacing (bcl 130–140 ms) the increase was smaller (Figure 4A), which can be explained by low levels of alternans throughout the tissue (Figure 3A). During rapid pacing (bcl 80–90 ms), even the non-infarcted zone manifested alternans, making the BZ/NZ alternans ratio approach 1. An extreme case of this is the single trace with BZ/NZ ratio of approximately 0.5 for high pacing frequencies (Figure 4A, arrow at bottom left), which was due to a special distribution of nodal lines in the border zone (Supplementary Figure S3).

The mechanism of calcium-driven alternans is usually considered to stem from insufficient calcium reuptake (Pruvot et al., 2004), which would manifest as a prolonged calcium transient. We therefore measured the average calcium transient duration at 90% calcium recovery (CaTD90, pacing at 140 ms bcl during which no alternans occurred) in all hearts. CaTD90 was significantly prolonged in border zone compared to non-infarcted zone tissue (*p* = 0.008, paired Wilcoxon signed rank test; Figure 4B), suggesting a dysfunction of calcium cycling in the border zone, thus making it more prone to alternans. Sham hearts manifested significantly shorter CaTD90 than both the non-infarcted and border zones of infarcted hearts (*p* = 0.012, unpaired Mann–Whitney *U* test; Figure 4B), suggesting that more efficient calcium cycling contributes to the resistance of sham hearts to alternans.

In cases of discordant alternans, several recordings manifested an obvious spatial correspondence between the edge of the border zone and nodal lines (Figure 4C,D,F,G), consistent with the hypothesis on structure-based location of nodal lines (Garfinkel, 2007). However, in other cases, no such relationship was apparent (Figure 4E,H), suggesting a different cause of formation of nodal lines, such as via calcium diffusion and fluctuation (Sato et al., 2013), or dynamic origin of nodal lines perpendicular to the direction of propagation (Garfinkel, 2007). Given that in several recordings we observed rather complex structure of nodal lines without any obvious perpendicularity to the direction of propagation (example in Figure 4E), the calcium diffusion and fluctuation hypothesis is a more likely explanation. Alternatively, it is possible that cardiac microstructure going beyond the macroscopic structure of the infarct scar is presenting a functional obstacle allowing formation of a nodal line.

### β-Adrenergic Receptor Stimulation Attenuates Alternans

**FIGURE 5:**
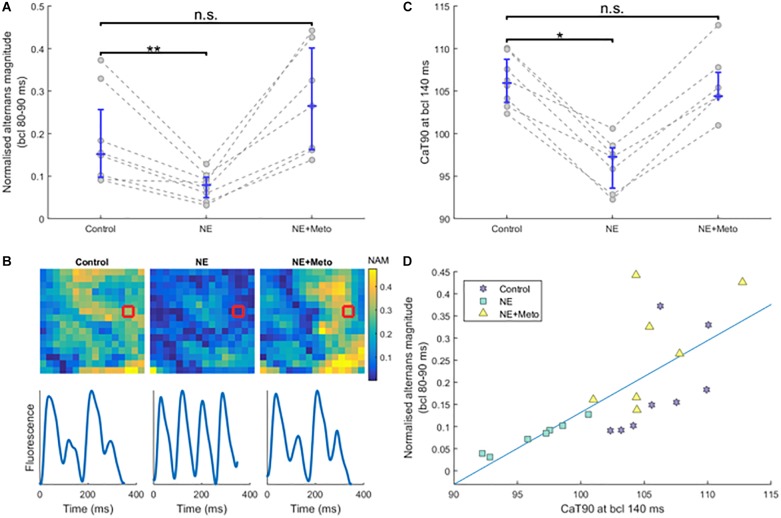
Effects of norepinephrine and subsequent β-blockade. **(A)** The effect of norepinephrine perfusion and subsequent β-blockade on normalized alternans magnitude at high pacing frequencies (each circle is an average of normalized alternans magnitudes of the given heart over bcls of 80, 85, and 90). Asterisks represent statistical significance at the level of 0.01 (^∗∗^), as assessed using the Wilcoxon signed rank test; n.s., not significant. **(B)** Sample alternans maps from a single recording under the three conditions (control, norepinephrine, and norepinephrine+metoprolol), with sample smoothed traces of the red-marked-pixels shown below. NAM, normalized alternans magnitude. **(C)** The effect of norepinephrine and subsequent β-blockade on CaTD90 (calcium transient duration, at 90% calcium recovery level) at pacing of 140 ms bcl (statistical significance assessed using the Wilcoxon signed rank test; ^∗^ corresponds to *p* < 0.05). **(D)** Correlation between CaTD90 from **(A)** and normalized alternans magnitude at fast pacing frequencies from **(C)**, matched by heart and condition. See Supplementary Figure S5 for a separation of **(A,C)** by normal and border zone.

In order to evaluate the effect of β-adrenergic receptor stimulation on alternans, we perfused norepinephrine, the main sympathetic neurotransmitter to the heart. The specificity of its effect was confirmed by subsequent perfusion of the β-blocker metoprolol. Norepinephrine potently attenuated alternans in hearts at rapid pacing intervals (80–90 ms bcl, *p* = 0.008, median 60% reduction in alternans; IQR 52–65% reduction) and this effect was blocked using metoprolol (Figure 5A,B); full frequency-dependent plots are given in Supplementary Figure S4. The attenuation of alternans with norepinephrine and its reversal with metoprolol were similar in non-infarcted and border zone regions, validating that the total effect is not driven by one region only (Supplementary Figure S5).

Norepinephrine perfusion resulted in shortening of CaTD90 at pacing of 140 ms bcl in all infarcted hearts (*p* = 0.016 for control versus NE; Figure 5C), which is consistent with the established effect of β-adrenergic receptor stimulation on calcium transient duration. This shortening was reverted with metoprolol (*p* = 0.58 for CaTD90 control versus NE+Meto; Figure 5C). The reduction of calcium transient duration with norepinephrine was due to a reduction of time to peak of calcium transient (median reduction 0.75 ms, IQR 0.02–2.74) as well as the time from peak to 90% recovery (median reduction 8.58 ms, IQR 6.15–9.64).

Interestingly, CaTD90 at the bcl of 140 ms (not inducing alternans) strongly correlated with normalized alternans formation at 80–90 ms bcl (Figure 5D, *R*^2^ = 0.52, *p* = 1.66 × 10^-4^, using the regression slope test), providing further evidence that impaired calcium handling might be the primary driver of alternans in our experiments.

### Computer Modeling of the Effect of β-Adrenergic Receptor Stimulation in the Infarct Border Zone and Its Influence on Arrhythmogenic Mechanisms

We then conducted computer simulations to see whether our experimental observations also apply in a large mammal ventricular model given what is known regarding β-adrenergic receptor signaling. We then investigated the consequences of border zone β-adrenergic receptor stimulation with regards to arrhythmogenic mechanisms. Computer simulations offer a controlled environment in which to test the effect of specific modulators of arrhythmias with high spatio-temporal resolution, thus overcoming experimental limitations.

**FIGURE 6:**
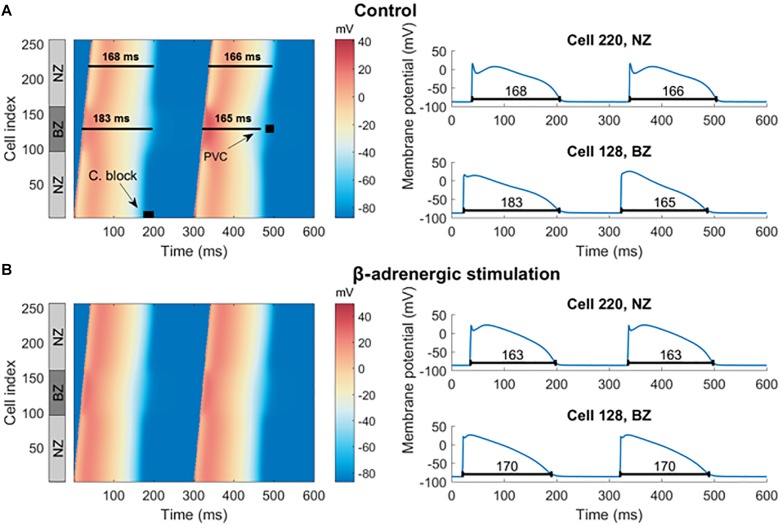
Pre-paced fiber at control condition and with simulated β-adrenergic receptor stimulation. **(A)** In the left half is a heat map of membrane potential of cells in the fiber over time, visualizing two action potentials manifesting alternans in the border zone. Every row of the image gives the membrane potential of a single cell in the fiber over time (the *x*-axis). The gray NZ/BZ/NZ box indicates the location of non-infarcted (NZ) and border (BZ) zones, with the numbers specifying the index of each cell in the fiber. The black rectangle labeled “C. block” indicates the segment of the fiber and the time of stimulation used in searching for the vulnerable window of conduction block. The black square labeled “PVC” shows the segment and time used in testing vulnerability to PVCs. To the right are examples of action potentials from NZ (cell index 220) and BZ (cell index 128), corresponding to single rows of the heat map. The black lines under action potentials specify the APD90. **(B)** An analogy of **(A)** with β-adrenergic receptor stimulation.

After 500 beats of pre-pacing of a cardiac fiber including a central BZ surrounded by non-infarcted cells, spatially heterogeneous APD alternans was present, restricted to the border zone virtual cardiomyocytes (Figure 6A). Simulation of β-adrenergic receptor stimulation abolished alternans in the border zone segment (Figure 6B), which reproduces our experimental results (Figure 5A). In both non-infarcted zone and border zone segments, β-adrenergic receptor stimulation also shortened the APD, as observed previously (Taggart et al., 2003).

**FIGURE 7:**
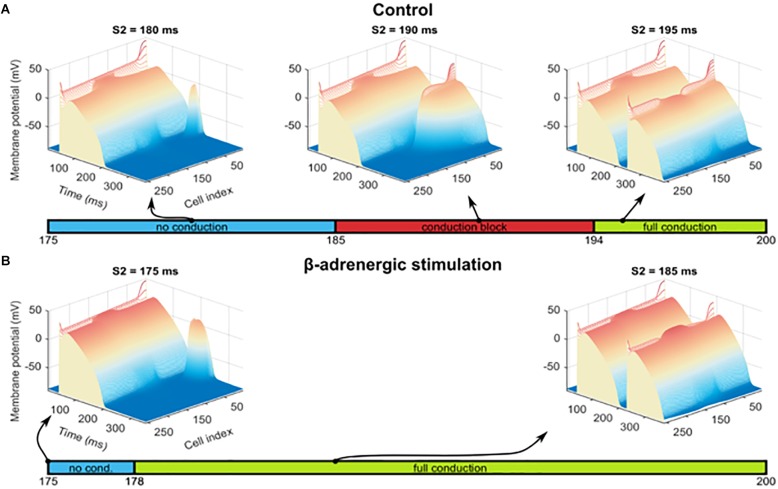
Alternans and conduction block. **(A)** Examples of three scenarios observed for S2 interval between 175 and 200 ms, with heat and elevation coding the membrane potential: no conduction of S2 stimulus beyond the stimulated segment of the fiber (left image), a conduction block in the BZ (central image), and full conduction through the fiber (right image). The bar under these three cases shows the S2 intervals in which these three conditions occurred. **(B)** Results of an identical protocol as in **(A)**, but using a fiber pre-paced in the presence of β-adrenergic receptor stimulation and manifesting no alternans. No conduction block was observed and thus is not shown.

In order to investigate other pro-arrhythmic consequences of β-adrenergic receptor stimulation in the border zone, we first tested the hypothesis that the prolonged APD during alternans would promote conduction block, a first step toward the establishment of re-entry. Thus, we quantified the vulnerable window for conduction block as explained in Section “Materials and Methods,” with S2 extra-stimulus applied at varying CI following the action potential with long APD during alternans in the border zone (“C block” label in Figure 6A) in both the control and the β-adrenergic receptor stimulated pre-paced MI fibers. In the control MI fiber, conduction block occurred in the border zone for S2 applied at CI = 185–194 ms (Figure 7A middle). For S2 applied at CI < 185 ms, no electrical propagation ensued as tissue was still refractory (Figure 7A left), while stimuli applied at CI > 194 ms propagated through the whole fiber normally as all tissue was fully recovered (Figure 7A right). β-Adrenergic receptor stimulation resulted in an increased window of vulnerability to conduction block following S2, as CI < 178 ms resulted in no propagation, and CI ≥ 178 ms resulted in full propagation through the fiber (Figure 7B). The increased window of excitability (S2 ≥ 178 ms compared to S2 ≥ 185 ms) under β-adrenergic receptor stimulation was due to the shortened APD and faster recovery of excitability (Figure 5B).

In contrast to the risk of conduction block associated with APD, we then hypothesized that the APD shortening in the opposite alternans phase might increase vulnerability to propagation of spontaneous depolarizations in cardiac tissue, via shortening the refractory period. This could enable an earlier re-activation following a premature ventricular complex (PVC) (Bogun et al., 2008). We thus computed the vulnerable window including all coupling intervals that lead to PVC propagation. To mimic a PVC, we applied a S2 stimulus at varying CIs in the border zone following the action potential manifesting APD shortening in the border zone (“PVC block” label in Figure 6A). Five different pre-paced fibers were compared (Table 2). In all cases, short enough S2 CIs resulted in no propagation (Supplementary Figure S6A), whereas longer S2 CIs led to propagation in both directions (Supplementary Figure S6B), with the difference between the fibers being in the minimum value of S2 allowing propagation (Table 2). Therefore, the vulnerable window is determined by the shortest coupling interval reported in the Table 2 along with the cycle length.

**Table 2:** Propagation of simulated premature ventricular complexes (PVCs) in several different myocardial fibers.

Fiber ID	Bcl (ms)	NZ/BZ composition	β-Adrenergic stimulation	Shortest CI with propagating S2 (ms)
A	300	NZ-BZ-NZ	0	176
B	300	NZ only	0	181
C	400	NZ-BZ-NZ	0	190
D	400	NZ only	0	184
E	300	NZ-BZ-NZ	100	180


*Bcl gives the pre-pacing cycle length (S1), NZ/BZ composition determines whether the fiber contains a BZ zone or is entirely composed of NZ cells, β-adrenergic receptor stimulation gives the relative concentration of β-agonist used, and shortest propagating S2 is the interval between the previous activation of the center of the fiber and the activation obtained using the S2 stimulus, representing the cells’ refractory period.*

First, we compared a fiber used in the previous section, manifesting alternans in the BZ (“fiber A”) with a fiber consisting of NZ cells only (“fiber B”), manifesting no alternans. Fiber A after the alternans-shortened AP was more vulnerable to premature activation than fiber B (minimum propagating S2 CI of 176 vs. 181 ms, Table 2 rows A, B), supporting the view that alternans might increase vulnerability to PVCs. In order to ascertain that this observation was due to alternans and not, for example due to a higher baseline vulnerability of the BZ, we repeated the measurements in fibers C and D, similar to A and B, respectively, but pre-paced at 400 ms bcl, manifesting no alternans (Table 2 rows C, D). We observed a lower vulnerability of fiber C to premature activation, compared to fiber D (minimum propagating S2 CI of 190 vs. 184 ms, respectively), showing that the effective refractory period of BZ cells is longer than in NZ cells, consistent with the literature (Wit et al., 1982). This is consistent with the hypothesis that the difference between fibers A and B is due to alternans, rather than baseline cellular properties. Ultimately, we measured the vulnerability to PVCs in fiber E; similar to fiber A, but with β-adrenergic receptor stimulation suppressing alternans, as in the previous section (Figure 6B). Fiber E was less vulnerable to PVCs than fiber A (minimum propagating S2 of 180 ms in E, versus 176 ms in A; Table 2 row E), showing a potential antiarrhythmic benefit of reducing alternans beyond a simple decrease in the risk of conduction block.

## Discussion

The main findings of this study are: (a) the healed infarct border zone is more prone to alternans than non-infarcted myocardium, predisposing hearts to spatially heterogeneous alternans, (b) β-adrenergic receptor stimulation attenuates border zone alternans in hearts with healed infarcts, and this can be modeled based on our knowledge of the effects of β-receptor signaling pathways on intracellular calcium handling and ion channels, (c) By reducing spatially heterogeneous alternans, β-adrenergic receptor stimulation also reduces the likelihood of conduction block and propagation of premature ventricular complexes in the border zone, both established contributors to the formation of re-entry. Together, these results help explain recent clinical imaging studies reporting that infarct border zone denervation predisposes patients to future arrhythmia episodes.

### The Infarct Border Zone Is Vulnerable to Alternans Formation

We demonstrate that hearts with a healed MI are prone to the formation of alternans (both in calcium transient amplitude and APD) consistent with previous work in humans (Koller et al., 2005). However, we also show that the border zone manifested stronger alternans than non-infarcted myocardium, predisposing the hearts to spatially heterogeneous alternans. Such spatial heterogeneity may promote conduction and repolarization inhomogeneities in a similar fashion as spatially discordant alternans, which is clearly linked to an increased risk of VF (Gaeta and Christini, 2012). In the infarcted hearts, we observed visible alternans in the border zone at frequencies within the physiological range of rat heart rate during exercise (Corre et al., 1976). This observation supports the importance of alternans in post-MI arrhythmogenesis, as alternans is reliably present at a given pacing frequency, unlike other drivers of VF, such as spontaneous depolarizations, which are comparably rare events. Interestingly, vulnerability to alternans at faster pacing rates (80–90 ms bcl) could be predicted from calcium transient duration at slower pacing rates (140 ms bcl), suggesting calcium oscillations as the alternans driver. A similar link between calcium transient decay dynamics at slow pacing and alternans vulnerability at rapid pacing was observed in the endocardium of a canine ventricular wedge preparation (Laurita and Rosenbaum, 2008). However, it is possible that the observed differences in alternans vulnerability in the study were linked to transmural heterogeneity beyond calcium transient dynamics. Our study used epicardial mapping in the whole heart, and the location of the mapping field was consistent between recordings, excluding the effect of base-apex gradients confounding alternans vulnerability.

### β-Adrenergic Receptor Stimulation Attenuates Infarct Border Zone Alternans in Post-infarction Hearts

In order to investigate the role of β-adrenergic receptor stimulation on alternans after MI, we perfused the sympathetic neurotransmitter norepinephrine, which considerably attenuated alternans magnitude (by 60 [52–65]% at fast pacing rates). This effect was prevented with the β-blocker metoprolol confirming β-adrenergic receptor stimulation as the causative factor. Computer simulations using a fiber model of canine ventricular myocytes reproduced these findings, based on our current knowledge of the effects of β-adrenergic receptor signaling pathways on intracellular calcium handling and ion channels.

### β-Adrenergic Receptor Stimulation in the Infarct Border Zone and Mechanisms of Arrhythmogenesis

We then explored how β-adrenergic receptor stimulation influenced other arrhythmogenic mechanisms in the border zone via its effect on alternans. APD prolongation as well as shortening during alternans is shown to be pro-arrhythmic, based on different mechanisms. First, spatially heterogeneous alternans restricted to the infarct border zone promotes conduction block after prolonged APD. This was present in the absence of remodeling of fast sodium current and gap junctions in the border zone, which may also promote conduction block in their own right. Conduction block is the first phase of functional re-entry (Wilson and Rosenbaum, 2007) and therefore alternans in the border zone may contribute to the establishment of re-entrant circuits in MI. Consistent with our experimental results, simulated β-adrenergic receptor stimulation abolished alternans, removing the increased risk of conduction block. This is in line with results from our previous single-cell computer model (Tomek et al., 2017), where acceleration in calcium release from the sarcoplasmic reticulum via β-adrenergic receptor simulation was identified as a key factor in alternans elimination.

Second, we propose a novel contribution of alternans to arrhythmogenesis via increased risk of propagation of pathological spontaneous ventricular depolarizations, during the shortened action potentials (Bogun et al., 2008). An important feature enabling this mechanism is the non-random formation of premature ventricular contractions (PVCs). For randomly occurring PVCs, the increased PVC propagation vulnerability for shortened APD would be outweighed by reduced propagation vulnerability for prolonged APDs. However, in many patients ectopic beats do not occur randomly through the cycle length, but occur preferentially early (Michelson et al., 1978). Therefore, during alternans when there is a shortened APD, the range of PVC coupling intervals which translate into ectopic waves would increase. An interesting feature of this pro-arrhythmic aspect of alternans is that it may be present during spatially concordant alternans, which has been considered largely benign compared to spatially discordant alternans (Wilson and Rosenbaum, 2007). However, during discordant alternans, the proposed phenomenon may be further facilitated, as an early afterdepolarization is more likely to be formed in the zone of a prolonged APD, which may then more easily propagate unidirectionally into the zone of shortened APD, promoting re-entry.

### Border Zone Denervation and Arrhythmia After Myocardial Infarction

There is controversy as to the role of localized sympathetic reinnervation (also called “nerve sprouting”) following initial denervation after MI, which restores β-adrenergic receptor stimulation to the infarct border zone. Multiple large-scale clinical imaging studies using catecholamine analogs have demonstrated that both the total volume of denervated myocardium and the volume of viable denervated myocardium following infarction predict ventricular arrhythmias and SCD (Boogers et al., 2010; Malhotra et al., 2015; Fallavollita et al., 2017). A study in isolated mice hearts also observed a lack of alternans in hearts with reinnervated infarct border zone due to tyrosine phosphatase σ gene knockout, although this was in the absence of either neuronal or β-adrenergic receptor stimulation (Gardner et al., 2015). Some of the beneficial results in this study could be explained by the potential pro-angiogenic action of tyrosine phosphatase σ inhibition (Ostman et al., 2006) aiding the revascularization of the border zone. On the other hand, a second stream of research, based mainly on animal studies (Shen and Zipes, 2014) claims that post-infarction nerve sprouting is pro-arrhythmic. These observations may be somewhat reconciled if excessive hyperinnervation provides similar amounts of repolarization heterogeneity as denervation. Regions of hyperinnervation observed in human transplant recipients with a history of ventricular arrhythmia were local findings at certain border zones (Cao et al., 2000b), suggesting a combination of denervation and hyperinnervation may occur, and this may be beyond the relatively low-resolution of meta-iodobenzylguanidine and 11C-meta-hydroxyephedrine PET imaging. Some, but not all aspects of this discrepancy may also be explained if the border zone innervation is dysfunctional (Lautamaki et al., 2015) or depleted of neurotransmitters (Correa-Araujo et al., 1991). Despite being structurally hyperinnervated in terms of nerve density, the innervation may functionally behave as if denervated. Interpreting studies is furthermore complicated by the fact that both increased and decreased function of β_1_ adrenergic receptors have also been observed in the border zone (Karliner et al., 1986; Gardner et al., 2015), and β_2_ receptor upregulation may compensate for β_1_ receptor downregulation (Lang et al., 2015). Given that it is currently impossible to simultaneously assess the functionality of regional innervation in relation to that of regional β-adrenergic receptor expression experimentally, we deliberately chose to focus our study on the responses to direct β-adrenergic receptor stimulation.

Our results are consistent with studies that report pro-arrhythmic effects of denervation, providing a novel mechanistic insight into post-infarction arrhythmogenesis. In the case of non-reinnervated border zone, two pro-arrhythmic factors seem to combine. The first aspect is the established observation that neural heterogeneity itself promotes electrophysiological conduction heterogeneity, which is pro-arrhythmic (Yoshioka et al., 2000), exacerbating the heterogeneity between non-infarcted zone and border zone. The second aspect, highlighted by our study, is the vulnerability of the border zone to electrical alternans, allowing the genesis of spatially heterogeneous alternans, which is pro-arrhythmic in a similar way to discordant alternans. Indeed, the combination of APD shortening in the innervated non-infarcted zone with APD prolongation in alternating action potentials could generate vast repolarization gradients. Sufficiently homogeneous reinnervation of the infarct border zone and subsequent β-adrenergic receptor stimulation of the innervated tissue then has the potential to both eliminate the pro-arrhythmic aspect of border zone denervation, but also to attenuate alternans, preventing arrhythmia via two distinct mechanisms.

It is noteworthy that while β-adrenergic receptor stimulation in the border zone might be beneficial via alternans attenuation in the setting of a healed MI, the situation seems reversed in the case of acute ischaemia where remodeling will not yet have occurred. T-wave alternans is exacerbated by sympathetic activation during acute ischemia (Nearing et al., 1991) and β-adrenergic receptor inhibition attenuates APD alternans in this setting (Murphy et al., 2017). Crucially, this study also reports that β-adrenergic receptor inhibition accelerates the decay of calcium transient, unlike in normal hearts or hearts with a healed MI, where β-adrenergic stimulation has this effect. Interestingly, given the opposite effect of β-adrenergic receptor stimulation on calcium transient duration in acute ischaemia versus healed infarction, the increased versus reduced alternans vulnerability follows naturally from the link between calcium transient duration and alternans vulnerability. Abnormal modulation of smooth endoplasmic reticulum calcium ATPase (SERCA) pump function during ischemia by β-adrenergic receptor stimulation may be a potential explanation for this. In these conditions, phospholamban seems to be fully phosphorylated, and β-adrenergic receptor stimulation thus cannot provide an increase in SERCA function. At the same time, β-adrenergic receptor stimulation promotes ATP consumption, on which the SERCA pumps directly depend, potentially leading to the net effect of paradoxical SERCA inhibition by β-adrenergic stimulation during ischemia.

### Limitations

While our model addresses the specific role of β-adrenergic receptor stimulation in the infarct border zone in an attempt to understand the results of clinical imaging studies, it does not attempt to recapitulate the intact *in vivo* system. It should be noted that cardiac autonomic control represents a multi-tier reflex control system with the potential to set up substantial heterogeneities in neurotransmitter release across the stressed heart, a fact that is exacerbated by reorganization of the neural projections into areas of myocardial damage. The isolated-perfused heart is devoid of these reflexes and norepinephrine infusion does not mimic the potential for neurotransmitter heterogeneity. Whereas norepinephrine causes uniform shortening in APD and dispersion of repolarization in the normal heart, sympathetic nerve stimulation increases heterogeneities in APD (Mantravadi et al., 2007; Ng et al., 2009; Yagishita et al., 2015). In addition, high levels of sympathetic nerve stimulation can cause the release of co-transmitters, such as neuropeptide Y, which may also modulate cardiac function (Tan et al., 2018).

In addition, no rodent model can fully recapitulate human acute MI (including atherosclerotic plaque rupture, platelet activation, and systemic inflammation), which is usually treated with prompt revascularization leading to smaller and less transmural infarcts. Coronary artery ligation is a commonly used model of MI, although we find it to be relatively unpredictable in terms of infarct size and transmurality and to be associated with high rates of mortality. In contrast, the more recently described cryo-infarction method produces a much more consistent size and location of injury with a lower mortality rate (Ciulla et al., 2004). Histological examination of hearts injured according to our method suggests a consistently high degree of transmurality and replacement fibrosis, and islands of viable tissue within the border zone as seen with coronary artery ligation. We find that cryo-injury causes a similar local inflammatory response to coronary ligation models (as shown in Supplementary Figure S7) and as expected causes adverse cardiac remodeling over the following weeks (Lewis et al., 2018). Others have also quantified the density of neuronal reinnervation in the border zone (Miwa et al., 2013) as observed in ligation models. All animal models are also confounded by the effects of general anesthesia and thoracotomy.

There may also be limitations relating to the optical mapping technique that we employ, although there is currently no other way of experimentally assessing calcium transient behavior across the myocardium. In particular the use of the mechanical un-coupler blebbistatin may disrupt the role of mechanical stretch from a PVC influencing APD dispersion or alternans. In addition, further information may have been gleaned from an additional camera to image a more zone remote from the infarct. We decided to focus on the interface between the border zone and non-infarcted myocardium to observe the potential for electrophysiological heterogeneity, but it is possible that even the “non-infarcted myocardium” underwent partial remodeling or included sparse micro-fibrosis and that there is further heterogeneity between our FOV and more remote myocardium.

### Clinical Perspective

The anti-arrhythmic benefit of β-blockers after MI and in congestive heart failure is firmly established in randomized controlled clinical trials (e.g., Cibis-Ii, 1999). Moreover, bilateral stellectomy results in a reduction in VT inducibility and correction in activation recovery interval (a surrogate for APD) in a porcine MI model (Irie et al., 2017). In patients with heart failure, refractory ventricular arrhythmia and failed catheter ablation, a large, contemporary series also demonstrates that bilateral cardiac sympathetic denervation resulted in a greater than 80% reduction in implantable cardiac defibrillator shocks following the procedure (Vaseghi et al., 2014, 2017). These interventions prevent β-adrenergic receptor driven intracellular calcium overload and reduce the chance of afterdepolarizations and PVC formation. They also counter the heterogenous shortening of APD and variation in conduction velocity which can predispose to re-entry even in the normal heart. In addition, by lowering heart rate they reduce cardiac metabolic oxygen demand, and improve coronary blood flow.

The potential benefit of infarct border zone reinnervation provides an interesting perspective on the arrhythmogenic role of the cardiac sympathetic nervous system: while ventricular infarct border zone innervation may be anti-arrhythmic via alternans attenuation, at the same time, a sympathetic-driven increase in heart rate via sinoatrial node innervation may be pro-arrhythmic, driving the heart into an alternans-vulnerable pacing window. This duality hints at a potential future therapeutic application via selective inhibition of the heart rate increase due to sympathetic stimulation, while maintaining the potential benefit of sufficiently homogeneous innervation in the ventricles and the respective peri-infarction β-adrenergic activity. Our observation that spatially heterogeneous alternans is a hazard in post-infarction hearts suggests that part of the anti-arrhythmic effect of β-blockers may also be by preventing an increase in heart rate toward an alternans-vulnerable pacing window. Moreover, β-blockers may cause hyperinnervation on their own (Clarke et al., 2010), and restore β-adrenergic receptor sensitivity (Heilbrunn et al., 1989), which our data suggests may help prevent alternans in the border zone. An interesting experiment would be to test whether β-blockers help reinnervate the infarct border zone, or at least prevent heterogeneity of innervation/β-adrenergic receptor stimulation. Given the controversy regarding the benefit of long term β-blocker use after MI without impaired LV function (e.g., Dondo et al., 2017), it would also be interesting to see whether our observations also occur in an MI model with little or no LV impairment where overzealous β-blockade could even be detrimental.

## Data Availability

All datasets generated for this study are included in the manuscript and/or the Supplementary Files.

## Author Contributions

JT, BR, GB, and NH contributed to the study design. JT, GH, AL, and CC contributed to the data acquisition. JT, MT, AL, CC, BR, GB, and NH contributed to the data analysis and visualization. BR, GB, and NH contributed to the funding and resource acquisition. JT contributed to the software. DP, BR, GB, and NH contributed to the supervision. All authors contributed to the writing. The authors approved the final version of the manuscript and are accountable for all aspects of the work in ensuring that questions related to the accuracy or integrity of any part of the work are appropriately investigated and resolved.

## Conflict of Interest Statement

The authors declare that the research was conducted in the absence of any commercial or financial relationships that could be construed as a potential conflict of interest.
