# Supplementary material for: β-Adrenergic Receptor Stimulation and Alternans in the Border Zone of a Healed Infarct: An ex vivo Study and Computational Investigation of Arrhythmogenesis
Source: Front Physiol. 2019 Mar 29;10:350. doi: 10.3389/fphys.2019.00350 (PMC6450465; doi:10.3389/fphys.2019.00350)
Supplement: Supplementary file 1 [file Data_Sheet_1.docx]

Supplementary Material

## 1. Supplementary Methods

## 1.1: Image processing methods

In this section, variable names are given in *italic* and the syntax *a*:*b*:*c* represents a vector ranging between *a* and *c* with step size *b*.

### Calcium alternans mapping

For each recording:

- The raw 32-by-32 pixel wide images are binned to 16-by-16 initially to improve signal stability and reliability. The *trace* is the time series representing fluorescence intensity over time for a given pixel. For each *trace*:
  - *trace* is linearly detrended and smoothed with Gaussian filter of width 20 (representing 20 ms).
  - The vector of local minima of *trace* is obtained, based on the base cycle length (*bcl*) of the recording, utilizing the fact, that the minima are spaced approximately *bcl* frames apart. First, for possible *starts* between 1 and *bcl*-1, vector of *start*:*bcl*:1000 ms is considered and the trace’s values at that locations are summed. The minimum over such sums is then taken, providing a good estimate of locations of minima and the respective vector of candidate minima is then used as seeds for the next step. There, each seed’s temporal neighbourhood with radius of 15 frames is searched to find an absolute minimum in the interval, which is stored for later use. The first and last minima are discarded as the calcium transients before the first one and after the last one may be incomplete.
  - For each pair of consecutive minima (with a calcium transient between these) and *subTrace*, the segment of trace between the minima:
    - *baseline* is estimated as the average of the trace’s values in those minima (these values may be different when alternans is present); *maximum* is the maximum of the trace between the consecutive minima.
    - Calcium transient amplitude is estimated as *CaTA* = *maximum – baseline* and calcium transient duration at 90% level is estimated as the length of *subTrace* segment above the threshold of *baseline* + 0.1 * (*maximum*-*baseline*).
  - Let *evenCaTAs* and *oddCaTAs* be the CaTAs of even and odd APs respectively. Then, normalized calcium alternans magnitude of *trace* and the respective pixel is defined as $\frac{|mean\left( evenCaTAs \right)-mean\left( oddCaTAs \right)|}{mean\left( evenCaTAs \right)+mean\left( oddCaTAs \right)}.$
  - Calcium transient duration associated with *trace* and the respective pixel is taken as an average of all durations of calcium transients in *trace*.
- The resulting maps of normalized alternans magnitude and calcium transient duration can be then spatially averaged to produce an average of the given recording, and/or split into non-infarcted zone values and border zone values using the manually obtained map of infarct border zone.
- For each pacing frequency and three conditions (control, norepinephrine, norepinephrine + metoprolol), there are three repeats available. When determining the alternans amplitude for a given condition and frequency, the three values obtained for the repeats are averaged to produce a single number.

During the imaging, three measurements were taken for a given condition (control/norepinephrine/norepinephrine + metoprolol) and bcl. When reporting total alternans magnitude, the three respective alternans maps are first spatially averaged to produce three numbers, which are then averaged to produce a single value per condition and base cycle length.

For visualizing the phase of alternans (Figure 3, main manuscript), we furthermore created annotations of the recordings encoding whether enhanced or diminished calcium transient was present at a given spatiotemporal position. For each pixel, if it manifests sufficient alternans (normalized alternans magnitude > 0.05), the frames during which it manifests enlarged calcium transient are marked with 1 and the frames during which it manifests diminished calcium transient with -1. Pixels with insufficient alternans magnitude are marked with 0 for all the frames. Thus, we obtain a stack of images consisting of {-1, 0, 1} and we can use its slices to assess in which phase which area is at a given frame. The slice used for Figure 3 of the main manuscript was taken at 500 ms after the start of the recording.

### Repolarisation alternans mapping

The generation of action potential duration (APD) maps from voltage mapping is broadly similar to the extraction of calcium transient amplitude alternans; however, the task is more challenging given unstable baseline of the voltage traces and the relative difficulty of extracting duration of an AP, compared to the amplitude. Voltage signals are more susceptible to residual tissue motion as RH237 fluorescence displays a small fractional change with each action potential compared to Rhod-2 fluorescence. Below are the steps of processing for a single trace:

- The trace is flipped up-down so that high membrane potentials are oriented upwards. Linear detrending and smoothing is performed as in calcium mapping, however, the smoothing filter width was reduced to 10 ms to preserve membrane potential upstroke.
- Vector of local minima is obtained as in calcium mapping, as well as vector of local maxima (the algorithm is identical, except that it works on –*trace* rather than the original one).
- For each local minimum, the value of local estimate of signal baseline is defined as the 10-percentile value within 15-frame radius of the given minimum (10-percentile was chosen over total minimum to reduce the effect of noise). Total *baseline* is defined as the average of those values. The estimate of maximum AP fluorescence, *peak*, is defined as the average over trace’s values in local maxima, which represent the highest points of each AP.
- The baseline estimates of the local minima are split into sets of even (*b_e_*) and odd ones (*b_o_*). As a quality control, traces with $\frac{std(b_{e})}{peak-baseline}>0.075$ or $\frac{{std(b}_{o})}{peak-baseline}>0.075$ are discarded (the respective mapping values set to NaN), as we observed the traces with such an unstable baseline to provide poor APD estimates. The split into even and odd baseline estimates is performed to represent the fact that at the fastest pacing rates, even the membrane potential baseline may oscillate under alternans and the increased standard deviation could thus be both due to instability or alternans, which is undesirable.
- For each segment of signal between two consecutive minima, representing an action potential, APD at any *level* scaled between 0 and 1 may be estimated as the longest segment above the threshold of *baseline* + (1 - *level*) * (*peak* – *baseline*).

The APD maps are then generated in a similar fashion as maps in the calcium mapping. The only difference is that the reference border zone/non-infarcted zone maps are flipped left to right to reflect that the voltage mapping FOV is a mirror image of the FOV of calcium mapping in our imaging setup.

## 1.2: Supplementary computational modelling methods

The single-cell model used in this study is based on our previous model of canine myocyte (Tomek et al., 2017), which is a fusion of the HeRd model of β-AR stimulation (Heijman et al., 2011) and infarct border zone remodeling data published by (Hund et al., 2008). The model includes the downstream effects of β-adrenoreceptor (β-AR) stimulation by arbitrary concentrations of the β-AR agonist isoproterenol (ISO). The currents affected by β-AR stimulation are: L-type calcium current (I_CaL_), phospholamban (PLB), slowly activating delayed-rectifier potassium current (I_Ks_), RyR, troponin-I (TnI), fast sodium current (I_Na_), sodium-potassium pump (I_NaK_), and ultra-rapid plateau potassium current (I_Kur_), as described in the original article. A diagram of the model is given in Figure S1, showing the currents and their regulation via CaMKII and β-AR stimulation. The model can reproduce, among other things, action potential adaptation, restitution, and accommodation, as well as β-AR-dependent changes in cyclic AMP levels, site phosphorylation and effects on electrophysiological properties, including APD and CaT amplitude (Heijman et al., 2011).

A non-infarcted cell may be switched to a border zone cell using changes published previously ((Hund et al., 2008)): increased CaMKII autophosphorylation rate (+425%), I_CaL_ (-36%), transient outward potassium current (I_To_; full block), background calcium current (I_Ca,b_ +33%) and time-independent inward-rectifier potassium current (I_K1_; -40%). Downregulation of INa in BZ cells in the original model was excluded in order to be able to allow us to attribute decreased excitability to alternans as opposed to a mixture of alternans and INa downregulation. The components remodeled in border zone are placed on yellow background in Figure S1. BZ cells maintain the original model of β-AR stimulation.

Three minor modifications were applied to the baseline single-cell model. First, a 25% increase in fast sodium current (INa) was included to compensate for electrical coupling of the cells in the fibre, allowing alternans formation (an identical modification has been used in (Tomek et al., 2017) when simulating concordant alternans in fibre). Second, a 35% reduction in expression of SERCA pumps in the BZ cells has been included, consistent with literature (Shi et al. 2015). Third, the modulation of inactivation and activation dynamics of INa under ISO stimulation included in the Heijman model was omitted (leaving an increased current amplitude by 25%); these modulations caused a considerable post-repolarisation refractoriness not consistent with literature showing that sympathetic stimulations shortens effective refractory period (Martins and Zipes, 1980).

Propagation of electrical excitation was simulated through one dimensional cardiac fibres consisting of 256 cells, being either all-NZ cells (control fibre), or with cells 96-160 being the BZ cells, used to study spatially heterogeneous alternans. All fibres were pre-paced for 500 or 501 beats (to have one state starting with a short action potential and one state starting with a long action potential), by stimulating an initial segment of the fibre with 1ms stimulus of -53 mV. A version of S1-S2 protocol (regular pre-pacing at S1 = 300 or 400 ms bcl, followed with a single stimulus, presented after S2 < S1 ms) was used to test the vulnerability to conduction block. This involves stimulating an initial segment of the fibre with a train of S1 stimuli, followed by a S2 stimulus at the same location. The window of vulnerability to conduction block is the range of S1-S2 coupling intervals (CI), where conduction stops in the infarct border zone. Similarly, a version of the S1-S2 protocol was used to test vulnerability to premature ventricular complex (PVC) propagation; however, in this case, the central segment of the fibre was stimulated with the S2 stimulus, mimicking a PVC. The S1-S2 coupling intervals reported in PVC testing are the interval between S1-driven activation at the central fibre segment and the activation induced via S2 stimulus. For each S1-S2 protocol, a range of possible CIs was explored: we re-loaded the pre-paced fibre after S1 stimulation for each tested S2 value.

The Myokit simulator was used to conduct the simulations, using its OpenGL GPU computing capabilities with a simulation time step of 0.002 ms (Clerx et al., 2016).

**1.3: References**

Clerx, M., Collins, P., de Lange, E., and Volders, P.G. (2016). Myokit: A simple interface to cardiac cellular electrophysiology. *Prog Biophys Mol Biol* 120(1-3)**,** 100-114. doi: 10.1016/j.pbiomolbio.2015.12.008.

Heijman, J., Volders, P.G., Westra, R.L., and Rudy, Y. (2011). Local control of beta-adrenergic stimulation: Effects on ventricular myocyte electrophysiology and Ca(2+)-transient. *J Mol Cell Cardiol* 50(5)**,** 863-871. doi: 10.1016/j.yjmcc.2011.02.007.

Hund, T.J., Decker, K.F., Kanter, E., Mohler, P.J., Boyden, P.A., Schuessler, R.B., et al. (2008). Role of activated CaMKII in abnormal calcium homeostasis and I(Na) remodeling after myocardial infarction: insights from mathematical modeling. *J Mol Cell Cardiol* 45(3)**,** 420-428. doi: 10.1016/j.yjmcc.2008.06.007.

Martins, J.B., and Zipes, D.P. (1980). Effects of sympathetic and vagal nerves on recovery properties of the endocardium and epicardium of the canine left ventricle. *Circ Res* 46(1)**,** 100-110.

Tomek, J., Rodriguez, B., Bub, G., and Heijman, J. (2017). beta-Adrenergic receptor stimulation inhibits proarrhythmic alternans in postinfarction border zone cardiomyocytes: a computational analysis. *Am J Physiol Heart Circ Physiol* 313(2)**,** H338-H353. doi: 10.1152/ajpheart.00094.2017.

**2. Supplementary Figures**


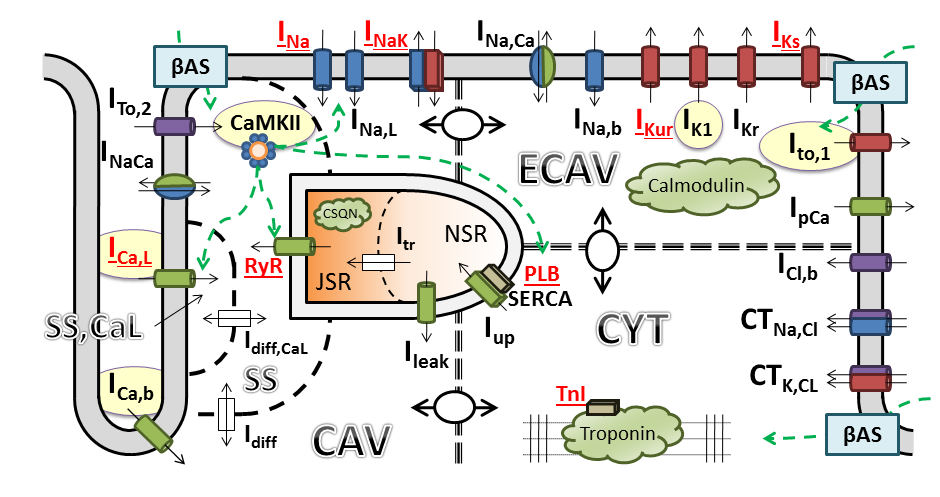


Figure S1: A schematic of canine myocyte electrophysiology, calcium handling, and signalling. In red are written components that are affected by β-adrenergic stimulation. The green arrows indicate activation via signalling, either by β-adrenergic stimulation, or CaMKII. Black letters with white border (e.g., "SS,CaL") indicate cellular subspaces, used to model different concentrations within the cell during an action potential. Adapted from (Tomek et al., 2017).


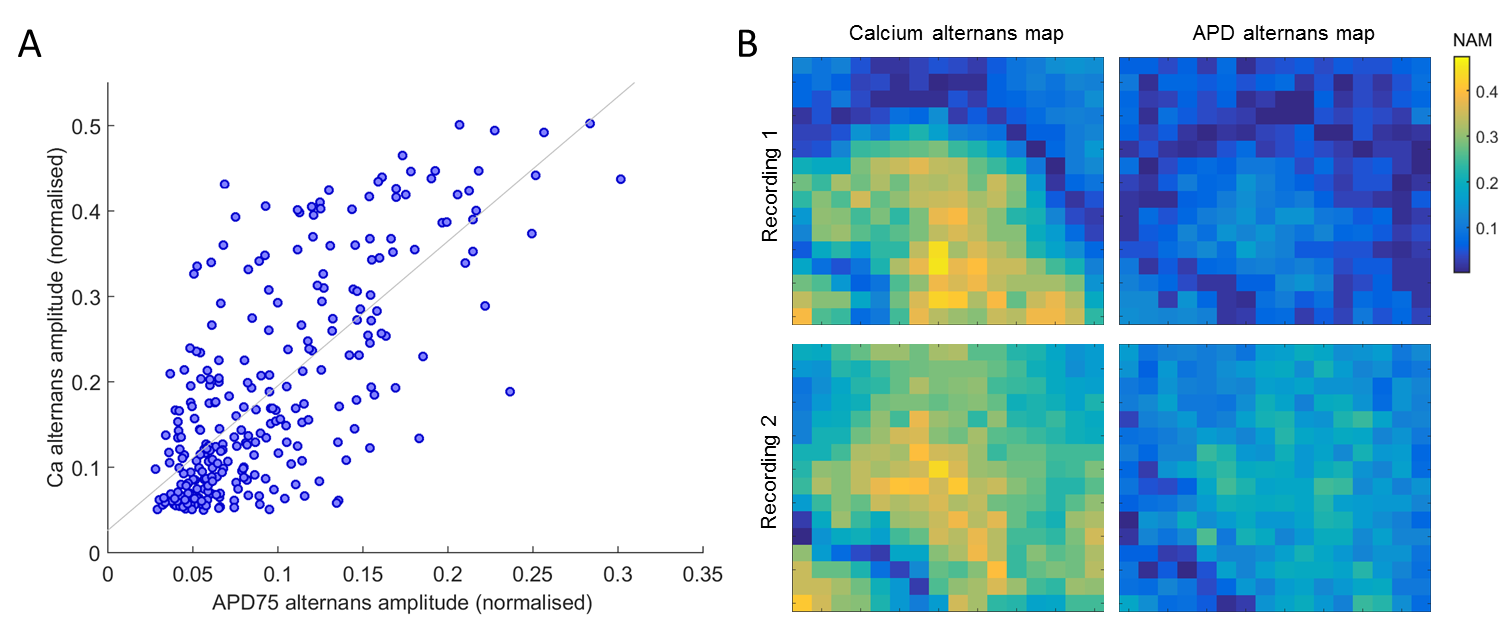


Figure S2: Calcium and APD75 alternans comparison. A: The correlation of normalised calcium and APD75 alternans amplitude per condition and base cycle length, which manifests Ca alternans amplitude > 0.05. B: Sample comparison of calcium and APD75 alternans maps from two recordings. The colour heat codes for normalized alternans amplitude (NAM), showing a good agreement of spatial distribution of alternans including the nodal lines. Normalized calcium alternans amplitude was clearly correlated with normalized APD75 alternans amplitude in the presented data (Figure S2A, p < 10^-47^ using slope test for correlation significance), typically including the spatial distribution of alternans within a single recording (FigureS2B). Given the good correlation of the two types of alternans and the lower stability of APD alternans estimation, we use only the calcium alternans mapping in the article. The lower accuracy of APD alternans estimation is chiefly due to a frequently unstable baseline in the voltage traces, resulting in the increased difficulty of estimating duration of an action potential compared to amplitude of calcium transient, which is a relatively simple task.


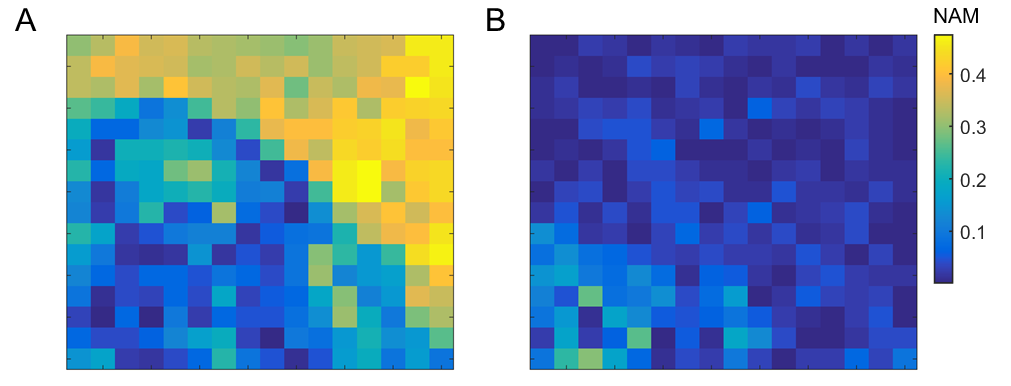


Figure S3: Singular case of low alternans in the border zone at rapid pacing. Shown are alternans maps of a single heart with the infarct border zone under the main diagonal of the map, at fast pacing (A; bcl = 85 ms) and at slower pacing (B; bcl = 120 ms). NAM = normalized alternans magnitude. While at slower pacing, the border zone tissue manifested clearly higher alternans than non-infarcted zone issue, this did not hold for fast pacing. There, the presence of nodal lines made the total border zone alternans low compared to the non-infarcted zone. Given the presence of nodal lines in the border zone and homogeneous alternans in the non-infarcted zone, this phenomenon does not necessarily imply lower arrhythmogenicity of the border zone.


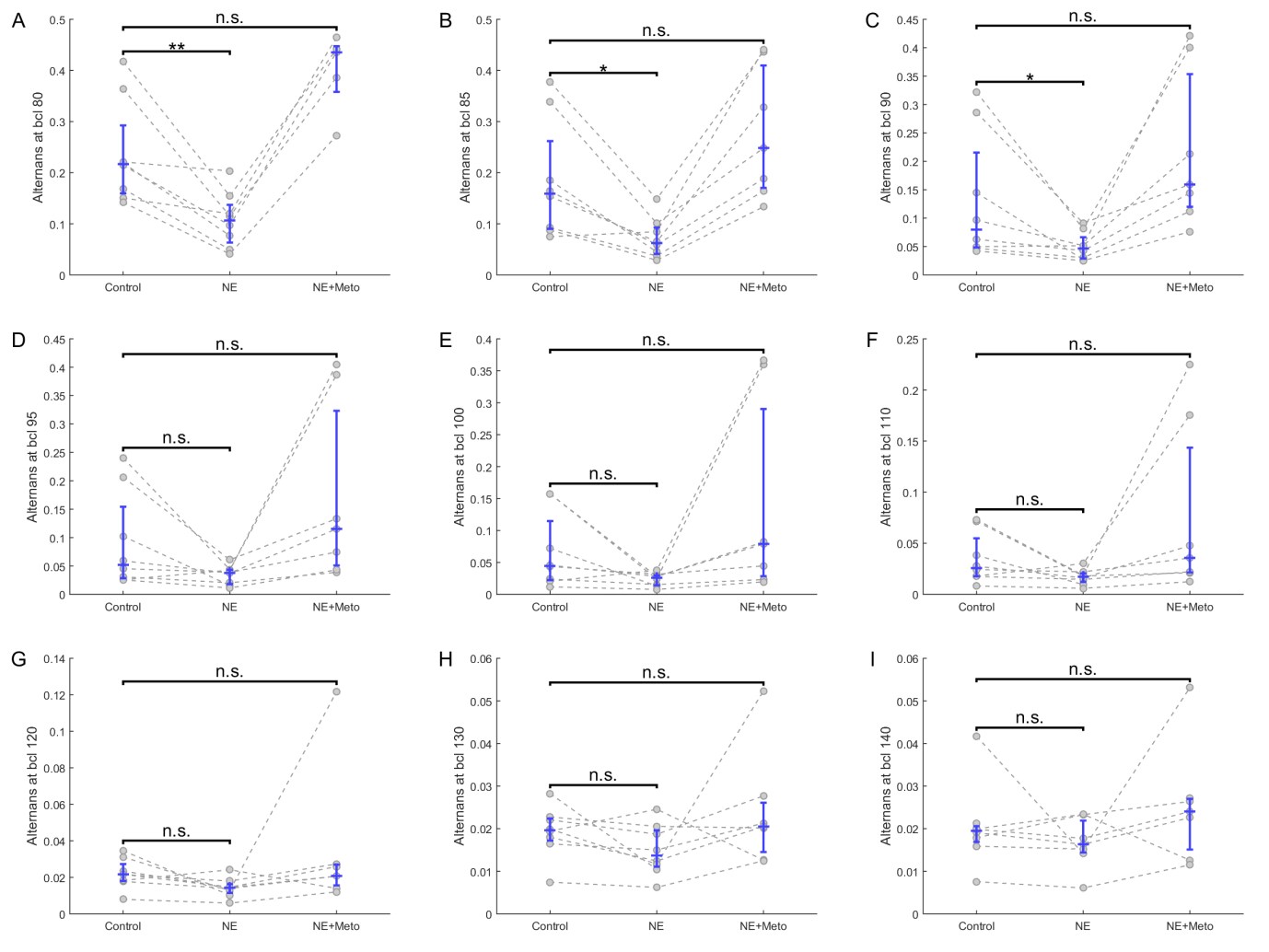


Figure S4: Normalized alternans magnitude for tested hearts at various pacing frequencies. The subpanels show normalized alternans at all the tested bcls (bcl is indicated on the y axis in each plot), showing that for each of three fastest pacing frequencies, norepinephrine (NE, 1 μmol/L) perfusion significantly attenuated alternans and this was reverted with the β-blocker metoprolol (10 μmol/L). For the other frequencies, alternans was attenuated with NE provided that the baseline alternans was large enough. The blue lines with whiskers show median and 25 and 75 percentiles. Asterisks represent statistical significance at the level of 0.01 (**), or 0.1 (*) using the Wilcoxon signed rank test.


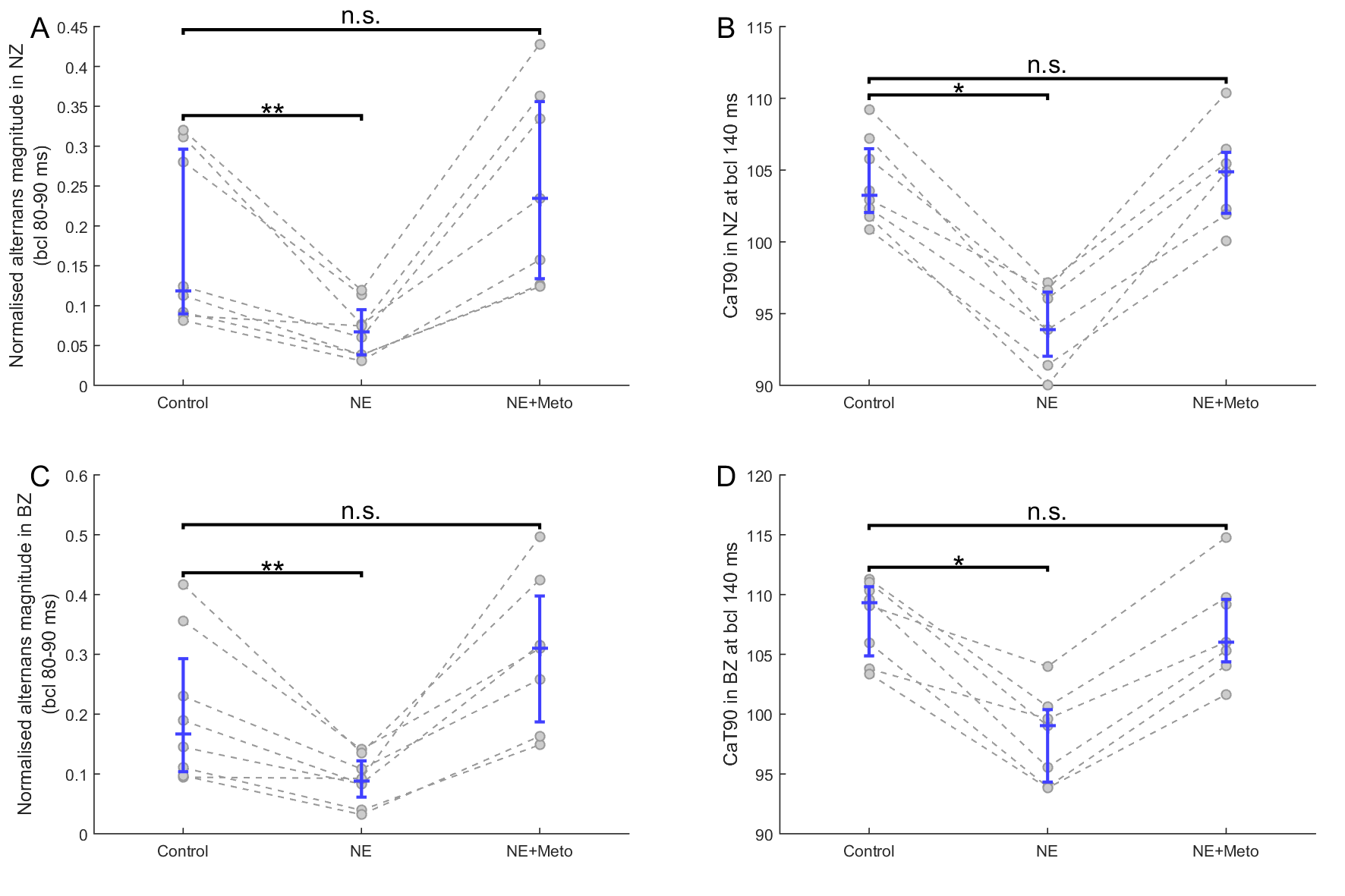


Figure S5: Norepinephrine attenuates alternans and shortens calcium transient duration both in NZ and BZ. A,B) are an analogy of Figure 4A,C of the main manuscript, using only data from the non-infarcted zone of each heart. C,D) are an analogy of A,B, but using only data from the border zone of each heart. Asterisks represent statistical significance at the level of 0.01 (**), or 0.1 (*) using the Wilcoxon signed rank test.


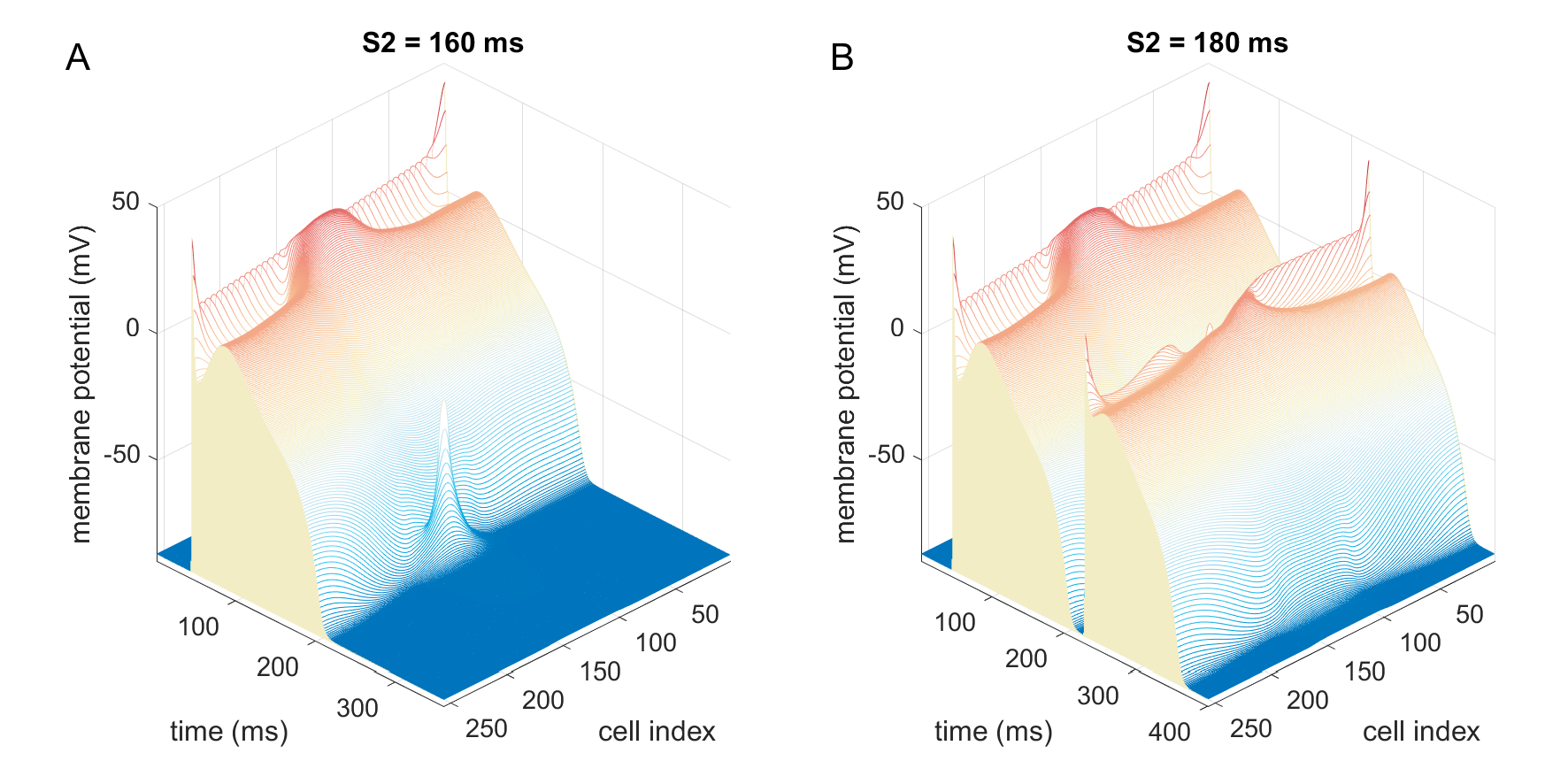


Figure S6: Propagation of simulated premature ventricular contractions (PVCs) in the border zone. A: An example of the lack of propagation of a stimulus to the BZ manifesting shorter of the two APDs in alternans, 160 ms after the previous activation of the stimulated segment. B: An example of a propagating stimulus, 180 ms after the previous activation of the stimulated segment; again, the stimulus was applied to the centre of the BZ, following a shortened APD.


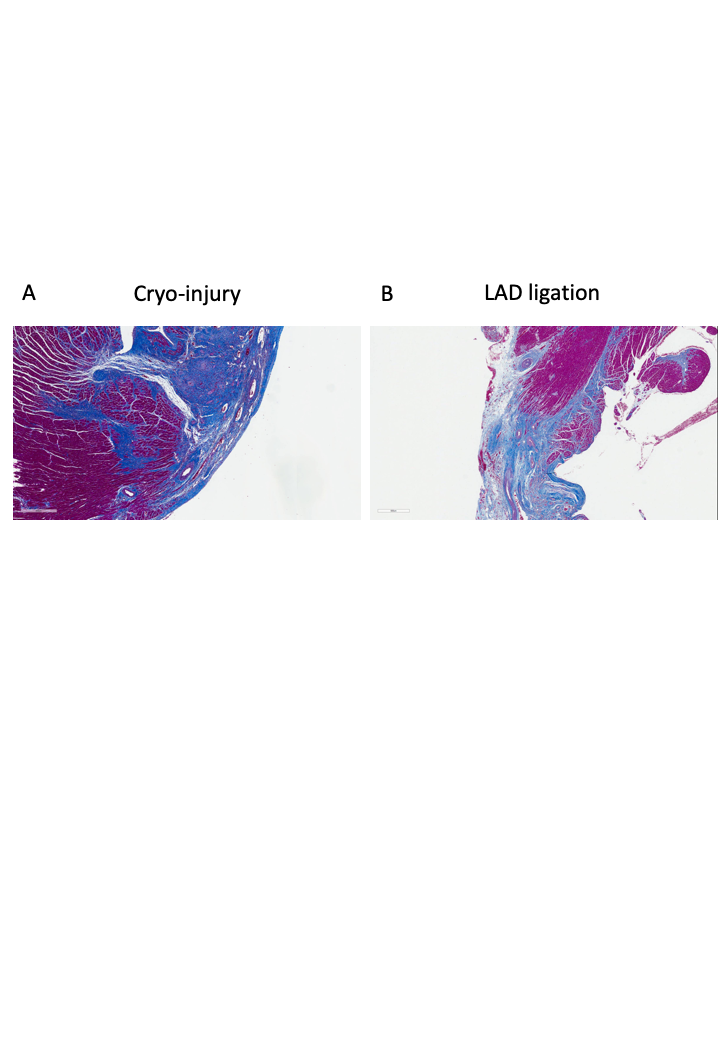
**Figure S7: Heterogenous scar substrate in the infarct border zone.** Example of Massons trichrome staining of the infarct border zone showing fibrotic scar formation and islands of viable tissue following myocardial infarction due to both **A:** cryo-injury and **B:** left anterior descending (LAD) artery ligation
